# Anatomy of an extinction revealed by molecular fossils spanning OAE2

**DOI:** 10.1038/s41598-021-92817-5

**Published:** 2021-06-30

**Authors:** R. M. Forkner, J. Dahl, A. Fildani, S. M. Barbanti, I. A. Yurchenko, J. M. Moldowan

**Affiliations:** 1 The Deep Time Institute, P.O. Box 27552, Austin, TX 78755-7552 USA; 2 Biomarker Technologies, Inc., 638 Martin Ave., Rohnert Park, CA 94928 USA; 3 Stanford University Institute for Materials and Energy Sciences, 476 Lomita Mall, McCullough Building, Stanford, CA 94305 USA; 4 Department of Geological Sciences, Stanford University, Stanford, CA 94305 USA

**Keywords:** Carbon cycle, Stratigraphy, Geochemistry

## Abstract

The Cenomanian–Turonian mass extinction (Oceanic Anoxic Event 2-OAE2) was a period of profound ecological change that is recorded in the sedimentary record in many locations around the globe. In this study, we provide a new and detailed account of repetitive changes in water column ecology by analyzing the organic geochemical record preserved within the OAE2 section of the Greenhorn Formation, Western Interior Seaway (WIS) of North America. Results from this study provide evidence that OAE2 in the WIS was the result of the cumulative effect of reoccurring environmental stresses rather than a single massive event. During OAE2, extreme variations in biotic composition occurred erratically over periods of several thousands of years as revealed by molecular fossil (biomarker) abundances and distributions calibrated to sedimentation rates. These cycles of marine productivity decline almost certainly had follow-on effects through the ecosystem and likely contributed to the Cenomanian–Turonian mass extinction. While the causes behind organic productivity cycling are yet unproven, we postulate that they may have been linked to repeated episodes of volcanic activity. Catastrophic volcanism and related CO_2_ outgassing have been interpreted as main drivers for OAE2, though this study provides new evidence that repetitive, punctuated environmental stresses were also important episodes within the anatomy of OAE2. Following OAE2, these cycles of productivity decline disappeared, and the WIS returned to conditions comparable to pre-OAE2 levels.

## Introduction

The Cenomanian–Turonian Boundary Event or Oceanic Anoxic Event 2 (OAE2) records a planet in crisis. Evidence includes: (1) isotopic excursions in carbon, oxygen, and sulfur^1–6^, (2) mass extinction of over a quarter of marine invertebrates^7,8^ (3) deposition and preservation of organic-rich sediments^6,9^, (4) a greenhouse climate with the Cretaceous Climatic Maximum starting in the Turonian^10^; and (5) enrichment of trace metals in marine sediments^4,6,11^. While it has been suggested that OAE2 may have been caused by multiple factors acting in concert, massive volcanic emplacement injecting CO_2_, H_2_S, SO_2_, and metal compounds into the ocean–atmosphere system has widely been interpreted as the main culprit driving OAE2^12–15^. Increased CO_2_ concentrations in the atmosphere and consequent heightened hydrologic cycle promoted runoff and increased nutrient flux, resulting in algal blooms and higher rates of organic carbon burial, leading to a globally-recognizable positive carbon isotope excursion (CIE) around OAE2^1,6,13,16^. The CIE is measured from both bulk carbon (carbonate rocks) and organic carbon (black shales) deposited across the Cenomanian–Turonian boundary^6^. Oxidation of trace metals, a decrease in ocean circulation related to higher world-wide temperatures, and the prevalence of shallow epicontinental seas led to severe water stagnation and acidification and is interpreted to have resulted large portions of the world’s oceans becoming anoxic (though the extent of anoxia varied locally)^17,18^. The prevalence of organic-rich layers preserved in pelagic marine paleoenvironments is used as supporting evidence for the global reach of the anoxic event^6,14^. However, deposits recording the Cenomanian–Turonian transition are not identical everywhere, particularly in shallower-water marine environments^6,9,10,16^. Variability in organic richness, thickness, lithology, and stratigraphic evolution through OAE2 indicate that at a minimum the “event” may be understood as a composite of multiple factors, including local inputs that left their imprint on the geologic record.

## Methods

**Figure 1:**
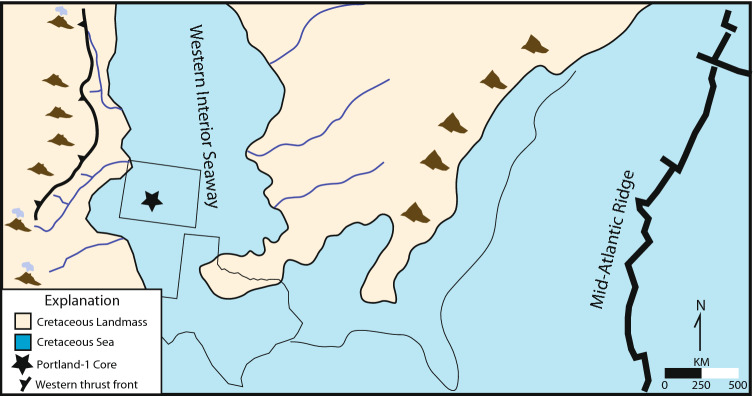
Paleogeographic map of North America during Oceanic Anoxic Event 2. The location of the Portland-1 core as well as active volcanic centers are shown. Map was drawn in Adobe Illustrator version 25.2.3 (www.adobe.com) from information presented in Caron et al. and Robinson Roberts and Kirschbaum^22,23^.

**Figure 2:**
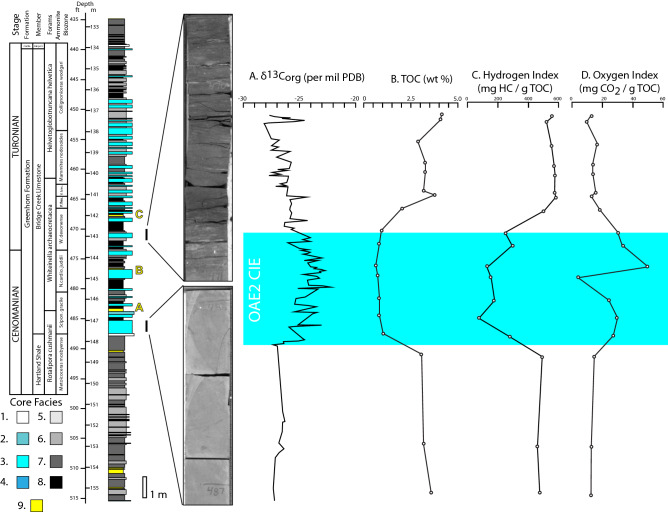
USGS Portland-1 core lithologic section, carbon isotope profile and RockEval data. Facies Explanation: 1. Peloidal/foraminiferal, packstone/grainstone; 2. Bioturbated peloidal packstone; 3. Bioturbated peloidal wackestone; 4. Skeletal grainstone; 5. Rippled mudstone; 6. Silty laminated mudstone; 7. Diffusely laminated mudstone; 8. Massive mudstone; 9. Bentonite. Samples were limited to facies 7 and 8. The occurrence of bioturbated peloidal carbonates during the OAE2 CIE indicates that the environment at the time of deposition was oxygenated and supported a diversity of tropical marine life. Note that the core is measured in imperial units as the Portland-1 core and core photos are curated with imperial measurements. This reference is preserved here in the case that the reader wishes to cross-reference these results to the Portland-1 core. Radioisotopic measurements from bentonites A, B, and C, along with biostratigraphy and correlation of depositional cycles to orbital timescales have produced an average sedimentation rate of 0.93 cm/kyr during the OAE2 CIE^19–21^. The interval of samples with the greatest flux in measured biomarker concentration occurs from ca. 473 feet to ca. 479 feet, in the central portion of the OAE2 CIE. Sample spacing in this interval is somewhat irregular in order to stay within the same depositional facies, but varies between 3 and 12 cm indicating that rapid flux in organic geochemical composition of analyzed sediments over periods approximately 3–15 kyr. The carbon isotopic excursion (CIE) that defines OAE2 is shown on the δ^13^C track and highlighted in blue on all compound tracks^20^. Hydrogen Index (HI) is generally negatively correlated with depositional environment oxygen concentrations, thus supporting the trend of OAE 2 CIE oxygenation. Oxygen Index (OI) generally correlates positively with depositional environment oxygen concentrations, and again provides evidence for oxygenation during the OAE2 CIE.

To garner new information about the biological conditions of the marine environment before, during, and after OAE2, we undertook a novel sampling campaign of organic material preserved in the USGS Portland #1 core, which includes the Cenomanian–Turonian Greenhorn Formation in Colorado, USA (Fig. 1). The nearly-continuous stratigraphic record of OAE2 was recognized by previous authors via the biostratigraphy, the carbon isotope excursion (CIE), and the radiometric age-dates from bentonites that define it temporally^19–21^ (Fig. 2A). Detailed core description informed sample collection through the OAE2 interval, as well as above and below the CIE. Sample collection initially targeted facies with higher amounts of reported TOC, and within which we could best determine low to absent amounts of reworking or bioturbation. This targeted sampling program limited sample collection to facies 7 and 8 and was done to ensure appropriate volumes of organic compounds could be extracted from samples for organic geochemical analyses^19,20^ (Fig. 2B). Because facies distribution is irregular, and also because our primary focus was the OAE2 CIE, sample spacing was dictated by the natural occurrence of target facies, and therefore is also somewhat irregular. Sample spacing varies from 3 to 12 cm within the OAE2 CIE, and 20 cm or more for samples outside the CIE. Individual samples were collected as ca. 1 cm thick by ca. 2–3 cm round sized pieces and then processed, first to determine richness and maturity (RockEval). Organic-rich samples were then high-graded for further analysis via GC–MS.

Molecular fossils or “biomarkers”, the recognizable remnants of biologically synthesized molecules found in organic-rich rock extracts and petroleum, were analyzed from organic extracts from our rock samples in order to obtain a series of snapshots of the water column ecology through OAE2^24^. As is standard, organic extracts were analyzed from and normalized to bitumen from the organic fraction. Based on this new molecular stratigraphy, (i.e., the presence or absence, relative abundance, and alteration of biomarkers contained within the core), it was possible to derive a wealth of new information with regard to the biota, depositional environment, and most importantly, the multiple drastic environmental changes that occurred before, during, and after OAE2 at this site.

Geological methods employed included standard lithologic description of core (including sedimentary and biogenic features, lithology, and texture) at the USGS core repository in Denver, Colorado, USA, and with use of core photographs where core was heavily sampled by previous workers. Sampling was carried out with the desire to compare equivalent facies and in order for analyses to track secular change. Sampling was biased to only include the same or similar facies, being the finest-grained dark mudrock that could be identified. This sampling scheme mitigated the effects of results being reflective of facies changes and increased the likelihood of recovering latent organic material.

Biomarkers were quantified by integrating compound peak areas and/or measuring peak heights relative to internal and external standards using parent-daughter ion transitions generated by analysis of branched and cyclic (B/C) fractions in an Agilent Technologies 7890A GC interfaced to a 7000A Triple Quad (GC–MS–MS). The external standard (Stanford-1) is a mixture of B/C fractions of several oil samples into which authentic synthetic biomarkers including 5β-cholane have been quantitatively added. 5β-cholane was also added quantitatively to the core extracts before they were separated into B/C and aromatic fractions. The response of each biomarker was determined relative to 5β-cholane in Stanford-1 at a specific MS–MS transition relevant for that compound and those calibration factors were used to determine the concentrations of each biomarker in each core extract. Results are reported as parts per million (ppm) concentrations or their ratios among multiple biomarkers.

## Results and discussion

### Oxic or anoxic?

Geological and geochemical data from the USGS Portland #1 core suggest that the water column of the Cretaceous Western Interior Seaway during OAE2 was not anoxic as the moniker ‘Ocean Anoxic Event’ might imply. At least at this location, direct sedimentological evidence from core (Fig. 2), confirms that the depositional environment of the water column/sediments was generally more oxygenated during the OAE2 CIE than it was before or after the event, which is consistent with many other studies from the area, as well as from other localities in the WIS, and time-equivalent shallow-water environments in the Tethys^16,19–21,25^. The transition from fine-grained clastic-dominated, laminated lithocycles before the event to extensively bioturbated carbonate-capped successions during the OAE2 CIE indicates a more biologically ‘healthy’ environment existed during OAE2 than before or after the CIE.

Investigating the organic geochemical record around OAE2 in the Portland #1 core confirms the findings of these geological analyses. First, Rock–Eval^P^ was used to determine the richness and thermal maturity of the organic material in the rocks being examined, and can also provide some insight into oxygenation of the paleo water column. Biomarkers require a measure of thermal maturity to develop, with the most desirable maturity for biomarker study being the very early oil window which is the case for this core (avg. T_max_ 435 °C, avg R_0_ = 0.6%)^24^. Rock–Eval “Hydrogen Index” (HI) is generally negatively correlated with depositional environment oxygen concentrations^26^. For this core, the samples taken above OAE2 had average HI values of 509 mg hydrocarbons/g TOC; within the OAE2 CIE the average was 177 mg hydrocarbons/g TOC; and below OAE2 the average was 423 mg hydrocarbons/g TOC, thus supporting the trend of CIE oxygenation (Fig. 2C). In addition, Rock–Eval^P^ “Oxygen Index” (OI) correlates positively with depositional environment oxygen concentrations. The average OI above OAE2 is 16 mg CO_2_/g TOC, within is 28 mg CO_2_/g TOC and below is 15 mg CO_2_/g TOC (Fig. 2D). We do note that there is a point of variability within the plotted oxygen index, and that this may indicate that there was some variability in oxygenation of the water column through the CIE. Such variability through the CIE would not be unexpected, as it is noted elsewhere^16,25^. Nevertheless, when compared to average measurements before and after the CIE, RockEval measurements are interpreted to indicate comparably higher levels of oxygenation in the water column during the OAE2 CIE than before or after the event.

**Figure 3:**
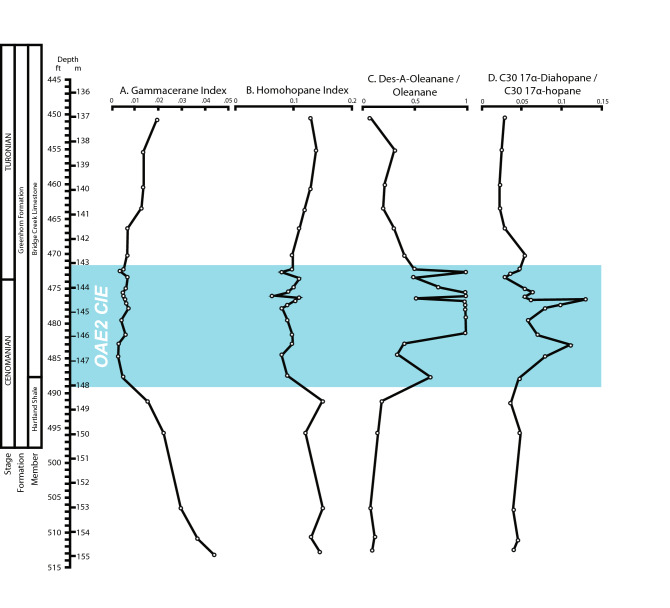
Compound tracks through OAE2 relating to oxygenation before, during, and after the event. Gammacerane and Homohopane Indexes, which are affected by sediment redox conditions, show a significant decrease and the ratios related to 17α-Diahopane exhibit an increase with striking fluctuations within OAE2. These broad scale changes reflect an overall increase in oxygenation during OAE2, with periods of reducing conditions punctuated through the event. The relative preservation of des-A-oleanane revealed by the des-A-oleanane/oleanane ratio could be a function of oxidation.

Several biomarker ratios have also been linked to water column and sediment redox conditions, and are likewise investigated here to decipher those conditions at the sample intervals (Fig. 3). The relative amounts of the biomarker gammacerane (likely derived from bacterivorous ciliates^27^) to bacterially-derived hopane, known as the Gammacerane Index, is usually associated with a stratified water column in source intervals, and high gammacerane is often interpreted to related to strongly reducing or hypersaline conditions^24^. In this case, the Gammacerane index decreases to its lowest measured values through the OAE2 CIE, suggesting that in a relative sense, the CIE was not on the average highly reducing in this locality (Fig. 3A). The ratio of C_35_ hopanes to the sum of C_31_–C_35_ hopanes, known as the Homohopane Index (HHI) is also generally observed to be high in reducing conditions in the marine realm and thought to be related to anoxia and/or increased bacterial sulfate reduction^24^. Again, in the case of the OAE2 CIE in this location, the HHI has its lowest measured values in the core, though some variability can be noted at the top of the CIE (Fig. 3B). Finally, both the concentrations of des-A-oleanane relative to oleanane as well as 17α-diahopane relative to 17α-hopane are evidence for more common periods of oxygenation during the CIE, with short intervals of reducing conditions present as well^28–30^ (Fig. 3C,D). This is because both des-A-oleanane and 17a-diahopane require some form of oxidation to be formed from their precursors (oleanene and hopane) in their source organisms (bacteria and flowering plants, respectively). While the concentrations of 17a-diahopane and des-A-oleanane are similar in the OAE to before and after the event the hopane and oleanane drop off, suggesting they were oxidized to lower levels. This means that in this case the ratios of des-A-oleanane to oleanane and 17α-diahopane relative to 17α-hopane can be taken as a measure of oxygenation in this core. In this case, the organic geochemical evidence from these ratio tracks corroborates the geological interpretations that unlike many other parts of the world’s oceans, the water column at this site was on the whole more oxygenated during OAE2 than before or after the event, though important pulses of variability exist within the succession. These pulses of variability in oxygenation, which are most striking between 473 and 479 ft in the CIE, likely relate rapid productivity cycling that are discussed later.

### Primary productivity

**Figure 4:**
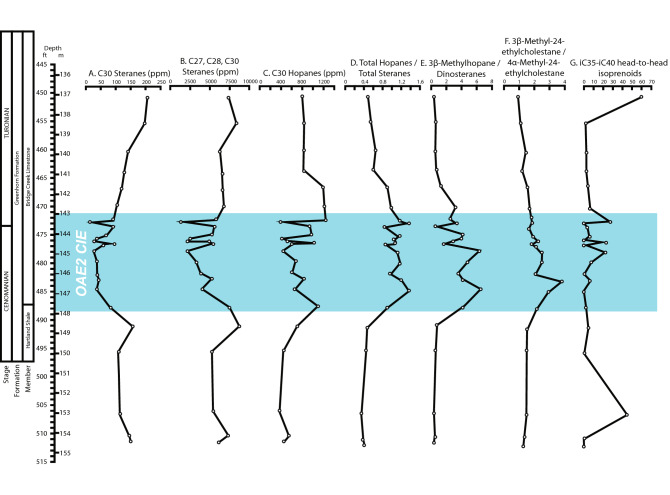
Compound tracks through OAE2 relating to productivity before, during, and after the event. For the main OAE2 section, the concentration of biomarkers derived from algae such as C_27_, C_28_, C_30_ steranes (24-*n*-propylcholestanes), and 4α-methyl-24-ethylcholestane 20R decreases significantly relative to concentrations of those derived from bacteria, which increase moderately with variations. This suggests that the record of productivity variability we interpret is reliable and is not simply a record of poor preservation of the organic fraction. Of note is the interval in all track from ca. ca. 473 feet to ca. 479 feet where organic geochemical measurements return the most erratic results. By applying the most recently published timescales through this interval, it is possible to calculate that the productivity cycles of biomarker decline and recovery can vary from ca. 26 kyr at the shortest to ca. 130 kyr at the longest. These productivity cycles occur within individual lithofacies internal to single lithocycles.

Measuring the biological indicators of marine productivity can provide an idea of ecological health during OAE2. Given that evidence for comparably healthy marine conditions exist at this site during the CIE (as opposed to before or after), the expectation was that there would be ample evidence for high primary productivity within the biomarker fraction. Biomarkers derived from algal primary producers include cholestanes, ergosteranes and C_24_-*n*-proplycholestanes, often referred to as C_27_, C_28_, and C_30_ steranes, together with saturated and aromatic dinosteranes derived from dinoflagellates^28,31–33^. Overall, concentrations of these biomarkers measured within the OAE2 CIE drop precipitously when compared to extracts from above and below the CIE (Fig. 4A,B). However, while there is a general trend of a drop in biomarkers within OAE2, the concentrations of these algal biomarkers also become highly erratic over short vertical distances within the core, especially toward the top of OAE2, between ca. 473 and 479 feet measured depth. Fluctuations in biomarker concentration occur at over 40% of total concentration several times through this interval. The biomarker concentrations related to primary productivity are measured from the same lithofacies, indicating repeated stressing of the paleoenvironment within the same depositional setting. These profound swings in biomarker concentration would otherwise be invisible to the geologist relying on lithological variability alone as a proxy for environmental change.

While steranes generally give indications of algal-derived organic input, hopanes are indicative of bacterial input^34–36^. Figure 4C,D shows that unlike steranes, the average concentrations of hopanes within the OAE2 CIE are fairly similar to extracts from above and below the CIE. However, like all biomarkers, concentrations of hopanes vary drastically (fluctuating several 10 s of percent relative concentration) within OAE2, indicative of the rapidly changing environmental conditions affecting the water column during the event. Similarly, large variations in the relative concentrations of 3β-methylhopane (a methanotroph byproduct) versus dinosteranes can be observed within the OAE2 CIE, and likely relate to variable redox conditions in the water column^28,37^ (Fig. 4E).

Erratic fluctuations in ratios of biomarker compounds edify the interpretation that primary productivity fluctuated significantly during OAE2 at this site. Three examples we highlight here confirm this interpretation. First, hopane/sterane ratios compare molecular fossils of both heterotrophic and photosynthetic bacteria to primary producers including marine algae and terrestrial plants. Second, 3β-methylhopane/dinosteranes compares 3β-methylhopanes (derived from aerobic methanotrophs and fermentative bacteria^37^) and dinosteranes, which are from primary producers, nearly exclusively dinoflagellates^31,38,39^. A third ratio is 3β-methyl-24-ethylcholestane (a reworked sterane)/4α-methyl-24-ethylcholestane (a marine algal sterane)^40,41^ (Fig. 4F). Importantly, tracks of these ratios indicate overall reductions in primary productivity during OAE2, but with drastic swings in ratios of several 10 s of percent concentration at a finer scale through the event. Rapidly changing biomarker ratios, coupled with rapidly changing biomarker abundances within the extracts, resulted from repetitive stressors acting on primary producers within OAE2. We call these rapid fluctuations in biomarker concentration “productivity cycles”, to differentiate them from observable lithocycles in the core, as they occur within a subset of facies inclusive of single lithocycles. Just as important as recognizing these productivity cycles in the core is recognizing where they do not occur: prior to, and following OAE2, we do not recognize evidence of this sort of productivity cycling within the organic fraction. Therefore, we interpret a link between the anoxic event and the occurrence of the productivity cycles recognized in the organic fraction.

The isoprenoids, pristane and phytane, are generally taken to be dominated by contributions from the chlorophyll side chains in phototrophic algae, although other precursor organisms such as cyanobacteria and certain archaea might be minor contributors^42–45^. Isoprenoids, in general, are low in concentration throughout the entire core, although there is some variability during the CIE (Fig. 4G). The head-to-head isoprenoids such as biphytane and its diagenetic products (bipristane), which are taxon-specific for marine archaea, occur only in trace abundances throughout the core and often drop to abundances below analytical detection limits^46,47^. Therefore, unlike previous work recognizing an abundance of opportunistic archaea during other Cretaceous OAE, there is no biomarker evidence of such an occurrence at this site^48^.

### Timescale estimate and rates of changes for environmental conditions within the OAE2

As described previously, biomarker abundances and their ratios change rapidly within OAE2 while they are relatively constant before and after the event. Both the USGS Portland #1 and other cores proximal to it have been the subject of extensive work to estimate sedimentation rates because of the prevalence of dateable bentonite ashfall beds in the succession^19–21^. Thanks to that work, an accumulation rate of 0.93 cm/kyr during the upper portion of the CIE was estimated by calibrating lithocycle stacking to both the latest orbital time scales tied to radiometric measurements from interbedded bentonites^19,20,49^. Utilizing this timescale gives a first order approximation of rate of change between different ecological states as indicated by organic geochemical measurements. The interval of samples with both the greatest variability and flux in biomarker concentration and ratios occurs from ca. 473 feet to ca. 479 feet (referenced in imperial units as the Portland-1 core and core photos are curated with imperial measurements), in the upper portion of the OAE2 CIE. This interval shows distinct fluctuations in the concentration of steranes (algae/primary producers) and hopanes. By applying this timescale, we calculate that whole productivity cycles of biomarker decline and recovery are both erratic and variable, occurring at ca. 26–ca. 90 kyr depending on the chosen cycle measured. Individual collapses can occur at our finest scale of sampling, which is 3 cm, or appx. 3.2 kyr.

### Hypothesized causes of rapid environmental changes

Influx of more oxygenated waters onto shallow, restricted epicontinental seaways, driven by modest sea-level rise, has been interpreted as one of the causes for the overall increase in oxygenation during the OAE2 CIE in the WIS^50,51^. Evidence of such a eustatic mechanism is also found in Tethyan localities where shallow marine carbonate facies transition from peritidal successions to successions dominated by deeper subtidal lithofacies during the whole of the OAE2 CIE^52^. However, while relief from stagnation might account for the overall increase in oxygen concentrations throughout OAE2 in the WIS, biomarker data presented here indicate that during OAE2, there were punctuated episodes of drastic change in environmental conditions that affected the ecology in the water column at a much finer scale. We struggle to refer to other studies that discuss biomarker variability this extreme within intervals this well-constrained, particularly any that are measured from within the same lithofacies. Because we observe this sort of drastic variability in organic composition within the same lithofacies, we surmise that whatever was driving the variability in biomarker composition during the CIE was not the same mechanism driving lithological change.

The most recent work to date in refining an astronomical age model to the Portland-1 core interprets ca. 100 kyr orbital eccentricity as the driver for the limestone-mudrock lithocycles observed in the area^49^. In addition, entire productivity cycles occur within individual lithofacies internal to single lithocycles, indicating that the driver of the productivity cycles is operating at a frequency different to, and higher than, that which drives lithological cyclicity. Because the productivity cycles we describe here are occurring at both rates faster than, and at more erratic intervals than the lithocycles, our current default interpretation of the biomarker data in this study is that it is recording aperiodic secular change external to currently interpreted drivers of lithological variability. While we cannot rule out the possibility of higher-frequency Milankovitch-band processes as possible influences on productivity cycles, both their erratic, irregular occurrence and the fact that they are not observed outside of the OAE2 CIE may indicate that their occurrence is related to the anoxic event itself.

We interpret the cycles in biomarker concentration in the Portland-1 core to have been the result of variability in primary productivity and subsequent organic matter decay. More “normal” times of productivity (e.g., before and after the CIE, and at like-productivity cycle intervals) correspond to more reducing conditions likely driven by higher rates of organic decay. This is in contrast to times of extreme stress, when primary productivity was suppressed as indicated by drops in biomarker concentrations and ratios. During periods of extreme stress, there was less organic matter in the water column leading to reduced rates of decay and higher relative oxygen concentrations. This variability is observed even though samples were recovered from like facies (dark mudstones) within individual lithocycles. In addition, other workers studying organic compounds from the OAE2 CIE in the WIS present data that also shows erratic distribution of biomarker compounds within the CIE, and even note that broad-scale productivity cycling likely occurred^53^. However, the cause of this productivity cycling, its erratic nature, and the fine time scales associated with the variability in concentration is left uninterpreted.

If the productivity cycles we observe in the biomarker data are not genetically linked to eustasy or orbital forcing, but instead punctuated periods of environmental stress with geologically rapid and erratic timescales, regional, episodic volcanism is a plausible hypothesis to consider. This is because the driver of environmental stress would have had to repeatedly and quickly disrupt primary algal production within the water column without also causing a complete change in the depositional environment that would alter the resultant lithofacies (as both eustasy and Milankovitch forcing are interpreted to have done). Indeed, several cores from the WIS (e.g., Eagle Ford, Boquillas, Bouldin Flags, etc.) contain numerous volcanically-derived bentonites at both the cm-and mm-scales, the analyses of several of the thicker of which provided age data from which sedimentation rates are calculated^19,21,25,31,49,51^. Possible factors such as regional sky-darkening related to repeated volcanigenic output or changes to the pH of the water column could be among the major drivers of marine productivity decline as recorded in biomarker composition-though admittedly such a hypothesis would require further testing to prove. In any case, these repeated episodes of productivity collapse and recovery occurred during OAE2, but are not detected before or after the CIE in this core. Clearly more comparative work should be done on the organic record of additional OAE successions to better understand the nature of water column biota through such events. Indeed, the occurrence of productivity cycles specifically within the organic geochemical record of the OAE2 CIE may indicate that multiple episodes of high-frequency environmental stress are a fundamental part of OAE2, and may challenge the notion that the OAE was wholly caused by a single catastrophic event.

## Conclusions

OAE2 was a period of profound ecological change globally. The geological record of that change varies from locality to locality, highlighting the influence of multiple factors-including local dynamics-controlling the paleoenvironmental conditions during the Cenomanian–Turonian transition. High-resolution organic geochemical analysis of a nearly-continuous core of sediment recovered from the Western Interior Seaway of North America shows that OAE2 was a time of unique and extreme environmental change. In the case of the WIS, the conditions just prior to OAE2 were comparably anoxic, with the OAE2 interval being generally more oxygenated than the moniker “OAE” would suggest. The conclusions from this study generally align with that view. In addition, organic geochemical analyses of organic extracts from core samples indicate extreme variability in biomarker concentration and composition during the OAE2 CIE. These cycles collapse and recovery occur internal to individual lithocycles, and were related to repeated ecological challenge of an unknown cause, though we hypothesize that they are related to repeated episodes of regional volcanism. Singular, catastrophic volcanic emplacement has been interpreted as the driver of OAE2 globally, including CO_2_ injection in the atmosphere, alteration of sea-water pH, high temperatures, and increased hydrologic cycle. However, the organic geochemical analyses in this study provide new evidence for multiple punctuated episodes of productivity collapse during the OAE2 CIE. During these episodes, marine biota was repeatedly challenged by high-stress conditions, the cumulative effects of which may have contributed to extinction within the broader ecosystem. Immediately after OAE2, no such organic variability is recognized within the data, and the system returned to comparable calm.
